# Natural Vascular Scaffolding Treatment Promotes Outward Remodeling During Arteriovenous Fistula Development in Rats

**DOI:** 10.3389/fbioe.2021.622617

**Published:** 2021-02-15

**Authors:** Yan-Ting Shiu, Yuxia He, Jason C. S. Tey, Marina Knysheva, Blake Anderson, Katalin Kauser

**Affiliations:** ^1^Division of Nephrology and Hypertension, University of Utah School of Medicine, Salt Lake City, UT, United States; ^2^Veterans Affairs Medical Center, Salt Lake City, UT, United States; ^3^Alucent Biomedical Inc., Salt Lake City, UT, United States

**Keywords:** arteriovenous fistula, extracellular matrix, neointimal formation, lumen expansion, dialysis, local drug delivery, photoactivation

## Abstract

Following creation, an arteriovenous fistula (AVF) must mature (i.e., enlarge lumen to allow high blood flow) before being used for hemodialysis. AVF maturation failure rates are high, and currently, there are no effective therapy to treat this problem. The maturation process is likely affected by the integrity of the vascular extracellular matrix (ECM). Natural Vascular Scaffolding (NVS) Therapy is a new technology that interlinks collagen and elastin via photoactivation of a locally delivered small molecule (4-amino-1,8-naphtalamide). We hypothesized that NVS Therapy may improve AVF remodeling by preserving ECM integrity. AVFs were created in Wistar male rats by connecting the femoral vein (end) to femoral artery (side) in the same limb. Immediately after blood flow was restored to dilate the femoral vein by arterial pressure, a 10 μl-drop of the NVS compound (2 mg/ml) was placed on the anastomosis perivascularly. Following 5-min incubation, the NVS treated area was exposed to 1-min illumination by 450-nm light. The control group received 10 μl-drop of phosphate buffered saline (PBS) and the same light activation. The skin was closed, and rats were euthanized 4 weeks (*n* = 6–9 per group) post-AVF creation for histology, morphometry, immunohistochemistry (IHC), and multiphoton microscopy for second-harmonic-generation evaluation of collagen fibers. The vascular thickness was similar in both groups. The AVF vein’s open lumen area and % open lumen area in NVS-treated rats were significantly larger than in PBS-treated rats (4.2-fold *p* = 0.014 and 2-fold *p* = 0.009, respectively). The inflammatory markers IL-6 and MMP-9 in the AVF walls were significantly decreased in the NVS group than the PBS group. Collagen fibers in the vascular wall trended toward perpendicular alignment to the lumen circumference in the NVS-treated AVFs, with more defined shape but less area than in the PBS-treated AVFs. These results indicate that the NVS Therapy exerted changes in collagen, which may influence AVF maturation. Rats tolerated the NVS treatment well, and the lack of cell death by the treatment was confirmed in cell culture experiments. These results suggest that NVS treatment is safe and may have therapeutic potential by facilitating lumen expansion to enhanced AVF maturation in patients.

## Introduction

Functional vascular access is a hemodialysis patient’s lifeline. Clinical practice guidelines elected an arteriovenous fistula (AVF), which is a direct connection of an artery to a vein in the upper extremity, as the preferred vascular access to provide adequate and efficient hemodialysis treatment to end-stage kidney disease (ESKD) patients (Lok et al., 2020). A newly created AVF needs to mature (i.e., grow to have a sufficiently large lumen to carry sufficiently high blood flow) in order to be used for dialysis. AVF maturation failure ranges from 20 to 60% in observational studies (Cheung et al., 2017). The high incidence of AVF maturation failure has recently prompted a re-evaluation of previous guidelines (Lok et al., 2020). Failure of AVF maturation, together with the rapid growth of the ESKD population, translates into increasing healthcare costs (Lee, 2017). Currently, there are no treatments available to enhance AVF maturation, and reliable AVF maturation represents an unmet medical need.

During the AVF maturation process, in response to the increase in wall shear stress and wall tension by the arterial blood flow, the venous wall undergoes adaptive lumen dilation and medial thickening to accommodate the changed hemodynamic forces. The desired outcome for successful AVF maturation is a timely and adequately arterialized vein via outward remodeling (Hu et al., 2016). Outward remodeling requires vascular wall thickening due to vascular smooth muscle cell proliferation *outside* of the internal elastic lamina (IEL) of the venous vessel wall, instead of *inside* of the IEL (i.e., inward remodeling) that may lead to neointimal hyperplasia formation and a decrease in lumen diameter (Hu et al., 2016). AVF maturation fails when inward remodeling is more dominant than outward remodeling, and hence the vein becomes stenotic. On the other hand, the outward remodeling process can be problematic if the vein dilates excessively and becomes aneurysmal. The molecular mechanisms involved are complex (Gorecka et al., 2019; Shiu et al., 2019; Lawson et al., 2020).

Vascular extracellular matrix (ECM) components and their regulators play an essential role in vascular remodeling in health and disease. However, it is not yet known whether an ECM targeted therapy may enhance AVF maturation. Natural Vascular Scaffolding (NVS) is a novel photochemical technology that utilizes a small molecular compound (4-amino-1,8 naphtalimide) that, upon activation by a 450 nm light, facilitates the reaction to create a covalent binding between amino acids of ECM proteins in the vascular wall. This local treatment takes about 5–6 min. It results in the cross-linking of collagen and elastin fibers of the vascular wall, thereby establishing a structurally stable, albeit still flexible, support frame for the vascular wall. The technology previously has shown in porcine carotid arteries to preserve the vessel’s natural integrity without increasing their stiffness (Mogharrabi et al., 2019) and to reduce overstretch-induced damage by balloon angioplasty (Munger et al., 2016). The NVS Therapy has been in clinical trials to investigate its safety and efficacy as a natural stent in patients with peripheral arterial occlusive disease [NCT04188262 (ongoing) and NCT03148808 (closed)]. It is conceivable that applying the NVS Therapy to the vein during AVF creation may protect from the hemodynamic change caused injury and hence promote AVF maturation via an ECM mediated mechanism. In the present study, the effect of NVS technology on AVF development was examined in a well-established rat model of AVF (Langer et al., 2010). We investigated the potential benefit of photochemical linking of ECM proteins in AVF maturation and the possible mechanisms contributing to this effect.

## Materials and Methods

### Microsurgical Procedure and NVS Therapy Treatment

**FIGURE 1:**
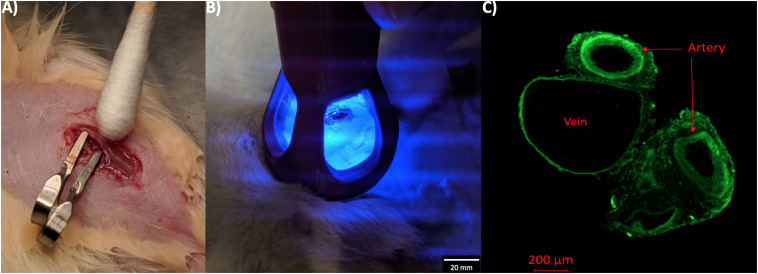
NVS treatment of AVF. **(A)** AVF surgery area was incubated with NVS solution for 5 min. **(B)** NVS was activated by laser at 450 nm for 1 min. **(C)** Fluorescent images of native blood vessels that were harvested immediately after the NVS and laser treatment. Green fluorescence indicates NVS penetration throughout the anastomosis area.

All studies and experiments were approved by the University of Utah Institutional Animal Care and Use Committee (IACUC) and performed in accordance with National Institutes of Health guidelines. All surgical procedures were performed on rats anesthetized by isoflurane inhalation (5% induction and 1–5% maintenance). Male Wistar rats of 10–12 months old (from Charles River Laboratories) underwent AVF surgery on the left femoral artery and vein as described in detail in the literature (Langer et al., 2010). Briefly, an end (vein) to side (artery) fistula in the same limb was surgically connected. Shortly after clamp removal, dilation of the vein occurred as a result of the arterial blood flow and pressure filling up the venous side of the anastomosis. At this time, a 10 μl-drop of the NVS compound (4-amino-1,8-naphtalamide) dissolved in phosphate buffered saline (PBS) at 2 mg/ml was applied perivascularly on the anastomosis, incubating for 5 min to allow the compound diffuse into the vascular tissue (Figure 1A). Following incubation, the small molecule treated area was exposed to 1-min illumination by 450-nm light source (Figure 1B). The control group received a 10 μl-drop of PBS and the same light activation. The skin was closed immediately after light activation, and then buprenorphine (0.05–0.1 mg/kg SQ) was administered immediately for pain relief. To confirm our NVS and laser treatment regime, two animals were sacrificed immediately after the 5-min NVS incubation and 1-min laser treatment, and their vessels were observed on a fluorescence microscope at 450 nm excitation for NVS (Figure 1C).

### Tissue Harvest and Fixation for Histology

Rats were euthanized at 1 week (*n* = 3–4 per group) or 4 weeks (*n* = 6–9 per group). The AVF vessels were harvested en bloc and placed in buffered zinc formalin for 48 h. The fixed tissues were embedded in paraffin and serially sectioned in 5 μm sections, following the standard histology procedures as previously described by us (Kwon et al., 2015; Shiu et al., 2016; Cho et al., 2020). Sections were stained with hematoxylin and eosin (H&E) and Verhoeff-Van Gieson (VVG) stain for subsequent morphometric analysis.

### Immunohistochemistry

Five-micrometer thin, unstained formalin-fixed-paraffin-embedded sections were put through a series of xylene and alcohol solutions to remove paraffin and rehydrate samples. Next, they were incubated in citrate buffer (pH 6.0, at 1X dilution, Abcam ab93678 Abcam) for 10 min for epitope retrieval, and incubated in hydrogen peroxide for 10 min first and then in blocking solution for 1 h to minimize non-specific binding. The treated slides were incubated with the primary antibody against matrix metalloproteinase (MMP)-2 (mouse monoclonal IgG, Abcam ab86607), MMP-9 (mouse monoclonal IgG, Abcam ab58803), interleukin (IL)-6 (rabbit polyclonal IgG, Abcam ab6672), or Ki67 (mouse monoclonal IgG, Dako M7240) overnight at 4°C. Dilutions used for antibodies were between 1:100 and 1:1,500. Sections were then incubated with the appropriate biotinylated secondary antibody (1:200, biotinylated goat anti-mouse or anti-rabbit IgG, Vector Laboratories BA-9200 or BA-1000, respectively) for 30 min at room temperature. The brown color was developed with ultra-sensitive avidin-biotin complex (ABC) peroxidase staining kit (ThermoFisher 32050) per manufacturer’s guidelines, and then with metal-enhanced 3, 3’ diaminobenzidine tetrahydrochloride (DAB) substrate (1X concentration, ThermoFisher 34065), followed by counterstaining with hematoxylin to give blue-gray color in the background.

### Image Acquisition

Whole-slide brightfield images of the H&E, VVG, or IHC-stained sections were acquired using ZEISS Axio Scan.Z1, under the same settings, at the University of Utah Cell Imaging Facility. The images were acquired at 5× for the lower magnification range and 20× for the upper magnification range, and scale bars were added to the whole-slide images.

### Morphometric Analysis and IHC Staining Quantification

In the VVG images, the IEL of each vessel was delineated using the freehand selection tool in the Fiji (an image processing package based on NIH ImageJ)^1^ to measure the IEL-enclosed area. The same method was used to measure the open lumen area [i.e., the area inside the lumen that is free of neointimal lesion (NL)]. The NL area was calculated as the IEL-enclosed area minus the open lumen area. The % area of open lumen was calculated as the open lumen area divided by the IEL-enclosed area and then multiplied by 100.

In the IHC images, the freehand selection tool in Fiji was again used to delineate the wall of the entire AVF, the AVF arterial limb, and AVF venous limb. The different colors in each IHC image were separated with consistent settings using the color deconvolution plugin in Fiji; the channel with the DAB signal was used. The DAB signal was subsequently highlighted with the threshold function; the intensity of the signal (expressed as mean gray value) and the % area of the vessel wall that stained positive were measured. The % area stained positive was calculated as the area of DAB signal in the vessel wall divided by the area of the vessel wall and then multiplied by 100. Sections where the primary antibody was omitted for negative control was used to subtract the background.

### Multiphoton Imaging of Collagen Fibers

Second harmonic generation (SHG) imaging was performed on 5-μm thick formalin-fixed paraffin embedded (FFPE) sections using Leica SP8 Dive with spectral tunable detection. Collagen fiber orientation in the medial layer was analyzed in relationship to the lumen. Each sample was divided into 4 quadrants and the medial layer was examined in each quadrant; the area of examination for each quadrant was 25 μm^2^. The same images were used for other collagen morphology using the MIPAR image software analysis.^2^

### Cell Culture

In *in vivo* experiments, animals were observed for toxicity. We performed *in vitro* experiments to investigate any toxicity effect of the NVS on endothelial cells and vascular smooth muscle cells, using cell death as a marker of toxicity. Human umbilical vein endothelial cells (HUVECs) were obtained from ATCC (PCS-100-013) and cultured in Medium 200 (GIBCO M-200-500) supplemented with 10% FBS (Hyclone Laboratories Inc., SH30071.03) and LSGS (GIBCO S-003-10). Human aortic smooth muscle cells (HASMCs) were purchased from ATCC (PCS-100-012) and cultured in medium 231 (M231-500) supplemented with 10% FBS (Hyclone Laboratories Inc., SH30071.03) and SMGS (S-007-25). HUVECs and HASMCs were maintained at 37°C in a 5% CO_2/_95% O_2_ atmosphere, and used between passages 4-8.

### Cell Death Assay

Cell death assay used the cell death detection ELISA kits from Roche (11544675001) and R&D systems (4822-96-K); both kits detect cell death via apoptosis. Cell density was 1 × 10^5^ cells per tub for the Roche kit and 1 × 10^5^ per well in a 96-well plate for the R&D systems kit. HUVECs and HASMCs were treated with NVS in different concentration (0, 0.25, 2.5, and 25 μg/ml) in serum free medium for 5 min in the incubator. After removing NVS, cells were washed with serum free medium once, and the plate was refilled with 200 μl serum free medium in each well. Cells were treated using 450 nm light for 1 min at room temperature, and then serum free medium was removed and each well was refilled with 200 μl of medium supplemented with 10% serum. At 0, 1, 2, and 3 days post-treatment (D0, D1, D2, and D3), cell death assay was performed according to the manufacturer’s protocol. Positive controls were cells incubated with hypertonic buffer (10 mM Tris PH 7.4, 400 mM NaCl, 10 mM MgCl^2^) at 37°C for 2 h.

### Statistical Analysis

GraphPad Prism was used to perform an unpaired, parametric t-test to determine the statistical significance of each of the parameters between the PBS-treated group and the NVS-treated group, with an alpha level set at 0.05.

## Results

### Morphometry of the AVF: The Entire AVF, the Venous Limb, and the Arterial Limbs

The pre-surgery IEL-enclose area in the vein was similar between the PBS and NVS groups (Supplementary Figure 1C, 32865 vs. 31499 μm^2^, *p* = 0.954). The pre-surgery IEL-enclose area in the artery was also similar between the PBS and NVS groups (Supplementary Figure 1D, 9060 vs. 7613 μm^2^, *p* = 0.703). At 1 week after AVF creation, both vein and artery areas significantly increased when compare to their pre-surgical values (Supplementary Figure 1), suggesting successful AVF creation surgery in both PBS and NVS groups.

**FIGURE 2:**
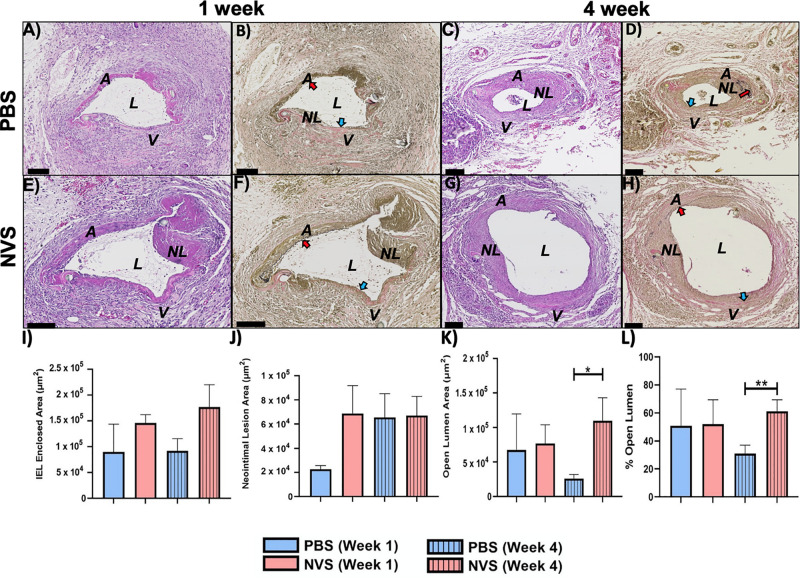
AVF anastomoses. Representative images of H&E **(A,C,E,G)** and VVG **(B,D,F,H)** staining of the AVF anastomoses at 1 week **(A,B,E,F)** and 4 weeks **(C,D,G,H)** from animals treated with PBS **(A–D)** and NVS **(E–H)**. **(I–L)** Morphometric analysis of the venous limb of the AVF anastomosis. Results are presented as average ± standard error of mean. *N* = 3–4 per group in week 1. *N* = 6–9 per group in week 4. *p < 0.05. **p < 0.01. Scale bar = 100 μm. A, arterial limb of AVF; V, venous limb of AVF; L, lumen; NL, neointimal lesion. Red and blue arrows indicate the internal elastic laminae of the arteries and veins, respectively.

Figure 2 displays representative histology images of the entire AVF and morphometric analysis of the venous limb at 1 and 4 weeks after AVF creation. Supplementary Figure 2 displays morphometric analysis of the entire AVF (top panels) and the arterial limb (bottom panels) at 1 and 4 weeks after AVF creation. Morphometric analysis was performed for four parameters: the IEL-enclosed area, the neointimal lesion area, the open lumen area, and the % area of open lumen. The IEL-enclosed area represents the maximal possible open lumen area without any neointimal lesion, because in a normal vessel the intimal layer is very thin, with just a single layer of endothelial cells (Alpers et al., 2017). We then compared the differences of these four parameters between the PBS and NVS groups at the same time point.

We first examined the entire AVF. We found that at 1 week after creation, these four parameters in the entire AVF were similar between the NVS-treated AVFs and the PBS-treated AVFs (*p* > 0.05). However, at 4 weeks, the NVS-treated AVFs exhibited a more expanded IEL-enclosed area (Supplementary Figure 2A, 1.6-fold, *p* = 0.200), open lumen area (Supplementary Figure 2C, 2.3-fold, *p* = 0.062), and % open lumen (Supplementary Figure 2D, 1.5-fold, *p* = 0.052) than the PBS-treated AVFs, though the trends were not statistically significant (*p* > 0.05).

We next examined, at week 4, the differences between the PBS and NVS groups at the venous limb vs. arterial limb. In the arterial limb at week 4, none of the differences in the four parameters were statistically significant (Supplementary Figures 2E–H). However, the venous limb of the NVS group had a 4.2-fold (*p* = 0.014) (Figure 2K) greater mean open lumen area and a 2.0-fold (*p* = 0.009) (Figure 2L) greater mean % open lumen area than the PBS group, even though both groups had similar neointimal lesion area (Figure 2J). These differences indicate that the NVS compound potentially improves AVF patency by improving outward remodeling in the vein.

### Immunohistochemistry of the AVF: The Entire AVF, the Venous Limb, and the Arterial Limb

Based on the results from the morphometric analysis, the expression levels of several molecular targets associated with AVF maturation failure (i.e., MMP-2, MMP-9, Ki-67, and IL-6) in the AVF vessels at the 4-week time point were selected for further investigation.

**FIGURE 3:**
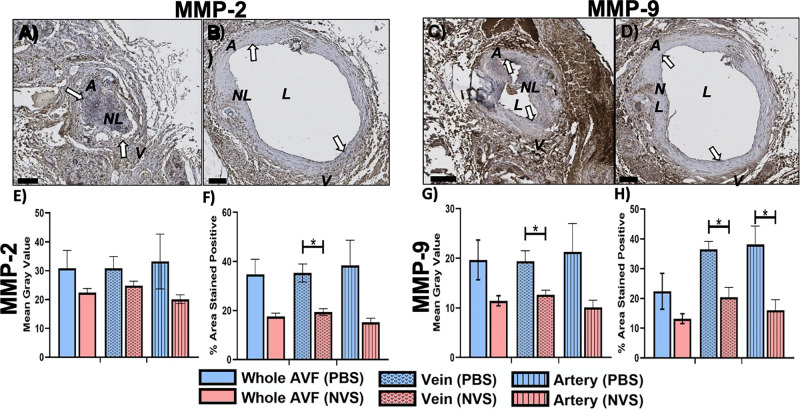
MMP analysis. Representative immunohistochemistry images of MMP-2 **(A,B)** and MMP-9 **(C,D)** staining of the AVF anastomoses at 4 weeks from animals treated with PBS **(A,C)** and NVS **(B,D)**. **(E,G)** Quantification of staining intensity (i.e., mean gray value). **(F,H)** Quantification of the percent area stained positive. Analyses were performed for the whole AVF, venous limb, and arterial limb. Results are presented as average ± standard error of mean. White arrows indicate example regions of positive staining. *N* = 3 per group. *p < 0.05. Scale bar = 100 μm. A, arterial limb of AVF; V, venous limb of AVF; L, lumen; NL, neointimal lesion.

Figure 3 displays representative IHC images and quantitative results of MMP-2 and MMP-9 analysis. The MMP-2 expression level was trending lower in the NVS group than in the PBS group in the entire AVF and the arterial limb; however, MMP-2 in the venous limb was significantly lower in the NVS group than the PBS group in terms of % area stained positive (Figure 3F, 1.8-fold, *p* = 0.015). In the entire AVF, the MMP-9 expression level was trending lower in the NVS group than in the PBS group; however, MMP-9 in the venous limb (Figure 3H, 1.8-fold, *p* = 0.019) and arterial limb (Figure 3H, 2.4-fold, *p* = 0.036) was significantly lower in the NVS group than the PBS group in terms of % area stained positive.

**FIGURE 4:**
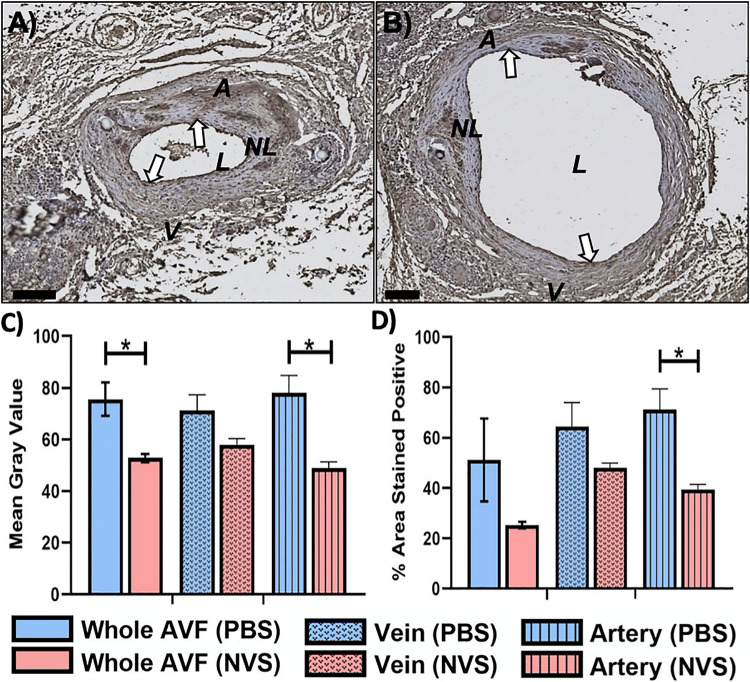
IL-6 analysis. Representative immunohistochemistry images of IL-6 **(A,B)** staining of the AVF anastomoses at 4 weeks from animals treated with PBS **(A)** and NVS **(B)**. **(C)** Quantification of staining intensity (i.e., mean gray value). **(D)** Quantification of the percent area stained positive. Analyses were performed for the whole AVF, venous limb, and arterial limb. Results are presented as average ± standard error of mean. *N* = 3 per group. *p < 0.05. Scale bar = 100 μm. A, arterial limb of AVF; V, venous limb of AVF; L, lumen; NL, neointimal lesion. White arrows indicate example regions of positive staining.

Figure 4 displays representative IHC images and quantitative results of IL-6 analysis. The IL-6 expression level was trending lower in the NVS group than in the PBS group in the venous limb. However, IL-6 in the entire AVF (Figure 4C, 1.4-fold, *p* = 0.027) and arterial limb (Figure 4C, 1.6-fold, *p* = 0.015) was significantly lower in the NVS group than the PBS in terms of mean gray value.

**FIGURE 5:**
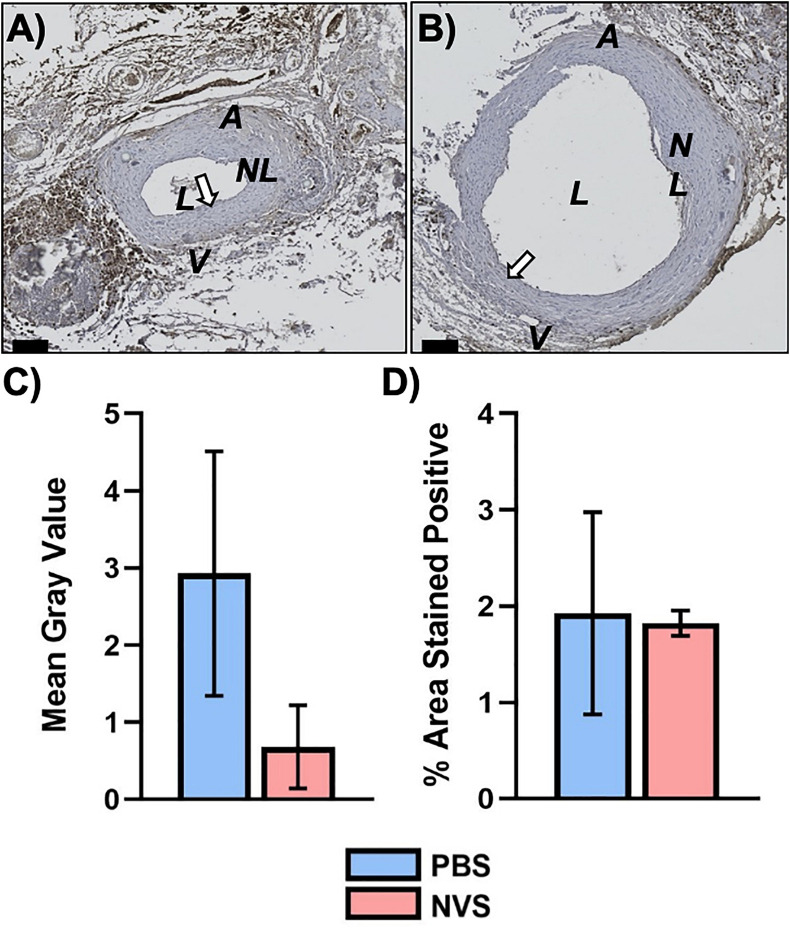
Ki67 analysis. Representative immunohistochemistry images of Ki67 **(A,B)** staining of the AVF anastomoses at 4 weeks from animals treated with PBS **(A)** and NVS **(B)**. **(C)** Quantification of staining intensity (i.e., mean gray value). **(D)** Quantification of the percent area stained positive. Analyses were performed for the whole AVF. Results are presented as average ± standard error of mean. N = 3 per group. Scale bar = 100 μm. A = Arterial limb of AVF. V = Venous limb of AVF. L = Lumen. NL = Neointimal lesion. White arrows indicate example regions of positive staining.

Figure 5 displays representative IHC images and quantitative results of Ki-67 analysis. The Ki-67 expression level was similar in the NVS group and the PBS group in the entire AVF.

### Multiphoton Analysis of Collagen Fiber Orientation

According to Martinez et al. (2018), collagen direction is considered favorable for AVF maturation if it is perpendicular to the lumen. We acquired SHG images of the AVFs at week 4 (Supplementary Figure 3). We observed that 58% of analyzed regions in the NVS treated samples exhibit favorable collagen orientation for AVF maturation, whereas only 25% of analyzed regions in the PBS treated samples exhibit a favorable collagen orientation, though this difference is not statistically significant. Collagen morphological data showed a trend for NVS treated samples to have less overall collagen area yet more defined shape features (i.e., roundness, roughness, and eccentricity) than PBS treated samples, although not statistically different.

### Cell Death Analysis

**FIGURE 6:**
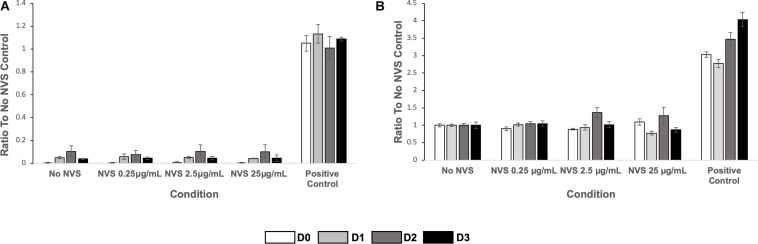
Cell death. **(A)** HUVEC apoptosis (left) and **(B)** HASMC apoptosis (right) of cultured cells upon exposure to NVS. Results are presented as average ± standard deviation.

As mentioned above, the animals tolerated NVS well and we did not observe any toxicity. To further confirm the absence of toxicity of the NVS concentration used, we performed *in vitro* experiments to measure the effect of NVS on the death of endothelial cells and vascular smooth muscle cells. NVS treatment caused minimal cell death via apoptosis, using the R&D systems kits (Figure 6) and Roche kits (data not shown).

## Discussion

Our study showed that rats tolerated NVS treatment well and the NVS treatment significantly increased the AVF open lumen area, without significantly affecting the neointimal hyperplasia area, suggesting that NVS treatment is safe and may have therapeutic potential by facilitating outward remodeling to enhance AVF maturation in patients.

Arteriovenous fistula is a surgically created conduit where an artery is directly connected to the vein. In response to the increase in wall shear stress and wall tension by the arterial blood flow, the venous wall undergoes remodeling to accommodate to the changed hemodynamic forces. The desired outcome for successful AVF maturation is a timely and properly arterialized vein via outward remodeling (Hu et al., 2016), but AVF maturation failure ranges from 20 to 60% in observational studies (Cheung et al., 2017). Improving AVF maturation remains an important clinical target for the management of ESKD patients (DeVita et al., 2020).

Arteriovenous fistula maturation failure is caused by luminal stenosis due to excessive neointimal hyperplasia and/or impaired outward remodeling. Among the many different biological processes, medial fibrosis has been highlighted in a recent paper as a previously overlooked, but critical contributor to poor maturation (Martinez et al., 2018). During the AVF maturation process, there is significantly increased expression of ECM components, regulatory proteins such as MMP and TIMP and structural proteins such as collagen and elastin, mediating a controlled pattern of ECM remodeling without structural failure (Hall et al., 2015). Excessive fibrosis of the wall due to local inflammation as well as the postoperative angle of collagen fibers showed positive association with AVF non-maturation (Martinez et al., 2018). In addition, the circumferential arrangement of the newly synthesized collagen was found to correlate with non-maturation of AVF, possibly due to decreasing the distensibility of veins as they go through the arterialization process of the vein. Proper ECM deposition can give structural and temporal direction for downstream biological processes contributing to vascular remodeling such as cell proliferation and inflammation (Del Monte-Nieto et al., 2020).

Therapeutic approaches targeting the ECM have been evaluated in the AVF setting previously. Topical administration of recombinant human elastase tested the hypothesis that degradation of elastin in the vessel wall may allow greater early dilation and the elastin fragments may have chemotactic properties redirecting migration of myofibroblasts thereby enhancing outward proliferation. Testing of this therapeutic principle in Phase II and III clinical trials has not shown benefit (Terry and Dember, 2013; Bleyer et al., 2019). Elastin is a non-regenerative component of ECM structural proteins. Facilitating elastin degradation likely causes more instability during arterial adaptation and that might have contributed to the lack of benefit instead of enhancing the maturation process.

Natural Vascular Scaffolding Therapy is a novel technology which helps to preserve the natural ECM scaffold using photochemical activation by a small molecule and 450nm light during the process of vascular dilation by angioplasty (Munger et al., 2016; Ansari et al., 2020). During photoactivation, the ECM fibers are relinked at a position they have been stretched to during the vascular procedure. Durable covalent bonds form between amino acids of collagen and elastin fibers, which help retain the enlarged lumen size, but remain flexible to pulsatile pressure unlike stent implants (Munger et al., 2016; Ansari et al., 2020). This scaffolding effect also helps to reduce the sudden hemodynamic impact by the changing blood flow during angioplasty procedures and indirectly contributes to the regulation of subsequent cellular responses. We have shown previously that the technology can preserve the natural integrity of porcine carotid arteries without increasing their stiffness (Mogharrabi et al., 2019), and can reduce overstretch-induced damage in porcine carotid arteries following balloon angioplasty (Munger et al., 2016). The treatment specifically targets ECM components and leaves the cellular vascular components functionally intact as it comes to contractility and relaxation (Ansari et al., 2020).

In the present study we have evaluated the effect of NVS Therapy in a well-studied rat model of AVF (Langer et al., 2010) to test the hypothesis that, relinking the ECM proteins at the time of AVF creation may lead to improved AVF development. Our results demonstrate that, when compared to the PBS control group, NVS Therapy did not affect the neointimal lesion area (i.e., no effect on inward remodeling) but, importantly, led to an enlarged venous diameter (i.e., better outward remodeling) by 4 weeks after AVF creation surgery. The similar Ki-67 expression level in the control and NVS groups is consistent with the notion that the NVS therapy does not affect the proliferation of vascular wall cells during the AVF development process. On the other hand, the increased lumen size was accompanied with decreased inflammatory markers IL-6 and MMP-9. A pathological role of inflammation in AVF maturation has been suggested in the literature. In particular, the first RNA-seq analysis of human veins harvested at the time of AVF creation surgery revealed that several pro-inflammatory genes were upregulated in pre-surgical veins that eventually failed to mature (Martinez et al., 2019). Additionally, IL-6 receptor activation was suggested to have a role in the pathogenesis of AVF failure in hemodialysis patients (Marrone et al., 2007). MMP-9 is an inflammatory marker; although MMP-2 is usually not considered an inflammatory marker, both MMP-2 and 9 have nearly identical subtracts and are important in vascular remodeling. The role of pre-surgical MMP-2/9 in AVF development is controversial. The pre-surgical, baseline levels of MMP-2/9 in serum and vein samples have been reported to have positive, negative or no association with AVF maturation in patients (Lee et al., 2010; Lin et al., 2010; Lee E. S. et al., 2011; Tirinescu et al., 2017). Work from our team suggested a negative association between MMP-2/9 and AVF maturation.

Our study shows that NVS does not cause cell death and does not affect cell proliferation during the AVF development process. Even though NVS has no effect on reducing inward remodeling, it still can benefit AVF maturation by enhancing outward remodeling. Stenosis at the juxta-anastomotic venous segment is common, and research efforts in the AVF field have focused almost exclusively on the role of neointimal hyperplasia formation as a cause of stenosis and subsequent AVF maturation failure (Dixon, 2006; Kokubo et al., 2009; Lee T. et al., 2011; Rothuizen et al., 2013). However, emerging clinical evidence suggests that stenosis alone cannot explain many cases of AVF maturation failure. For instance, in a study of 145 patients, two-thirds of AVFs with stenosis still matured without treatment of the stenosis (Allon et al., 2013). Another study of 115 patients found that post-operative neointimal hyperplasia was only associated with maturation failure when patients had more fibrotic and hence more rigid veins (Martinez et al., 2018). These observations strongly highlight the need for more research to enhance lumen expansion during the AVF development process, not just to inhibit stenosis due to excessive cell proliferation and neointimal hyperplasia formation.

Currently we do not yet know exactly how/why ECM relinking may lead to reduced inflammation markers, and this is beyond the scope of the present study. Similar observation has been made applying the treatment to diseased human arteries *ex vivo* (Ansari et al., 2020). In those studies, IL-6 production of human arterial rings was inhibited in organ culture following NVS Therapy treatment in comparison to control. These previous studies suggest a direct inhibition of inflammation by NVS, but further investigation is needed to understand the exact mechanism of the anti-inflammatory effect of NVS treatment.

Since the NVS Therapy primarily affects the arrangement of ECM, we have investigated the difference between collagen fibers in the NVS treated vessels in comparison to the PBS controls, which had also undergone the light activation treatment. Multiphoton SHG imaging is a useful technology to investigate collagen fiber organization; it takes advantage of the collagen’s optical property and does not require histological staining (such as Picrosirius red or Masson’s trichrome) or immunostaining using antibodies. Collagen fibers in the vascular wall trended toward perpendicular alignment with respect to the lumen circumference in the NVS-treated AVFs. This is in agreement with the findings by Martinez et al. (2018). Distribution and position of ECM fibers in the vessel wall may contribute to downstream biology. The scaffold provides the microenvironment for the cellular component in which proliferation and inflammation are regulated.

This study has several limitations. First, the animal numbers could be larger. Future studies should consider the use of larger cohorts of animals. Second, the vessel wall lumen area and the % area of open lumen were obtained from fixed tissue. It is well known that tissue will shrink after formalin fixation. In addition, unlike arteries, lumen collapse is commonly seen in harvested veins due to their thinner wall. Future studies will include the use of non-invasive ultrasound to measure lumen area in live animals to complement the histological findings. Third, we used IHC to assess MMP-2 and MMP-9 protein expression levels in the vessel wall. Both MMPs are secreted as inactive preproteins, which are activated when cleaved by extracellular proteinases; their activity levels are further regulated by tissue inhibitors of metalloproteinases (TIMPs). Thus, zymography would be a critical assay for determining the MMP activity levels in the vessel wall and should be considered in future studies besides the histological determination of their expression. Fourth, the mechanistic study was limited in scope and IHC was used, instead of more quantitative methods such as Western Blot or ELISA. The present study’s primary goal was to elucidate if there is an effect of NVS on AVF remodeling and therefore, morphometrical analysis of the lumen area was the most critical parameter. As the first step to investigate the underlying molecular mechanism, we performed IHC analysis and found that the inflammatory markers IL-6 and MMP-9 in the AVF walls were significantly decreased in NVS treated vessels. Future studies, potentially in large animal models (e.g., pig or sheep) will consider the use of more quantitative methods (e.g., Western Blot or ELISA) and pathways upstream to IL-6 and MMP-9 (e.g., reactive oxidative species and oxidative stress).

## Conclusion

Our data demonstrated that NVS Therapy when applied at AVF creation can promote outward remodeling of the venous wall, concomitant to reduced inflammation. These beneficial downstream biological effects by the NVS treatment correlated with the more distinct and measurable appearance of collagen. Our result supports previous conclusions based on clinical observations emphasizing the role and correlation of proper vascular ECM organization during the AVF maturation process. It also suggests the potential for NVS Therapy to enhance fistula maturation in the clinical setting.

## Data Availability Statement

The datasets presented in this article are not readily available because the raw data supporting the conclusions of this article will be made available by the authors to the requester, after establishing a Data Usage Agreement between the requester’s institute(s) and the University of Utah and a Data Usage Agreement between the requester’s institute(s) and Alucent Biomedical, Inc. Requests to access the datasets should be directed to KK, kkauser@alucentbiomedical.com.

## Ethics Statement

The animal study was reviewed and approved by University of Utah IACUC.

## Author Contributions

YTS and KK: study design and data interpretation. YH, JT, MK, BA: data collection. All authors: data analysis, drafting manuscript, and approving the final version of the manuscript.

## Conflict of Interest

BA and KK were employed by company Alucent Biomedical Inc. The funder Alucent Biomedical, Inc. was involved in the study design, collection, analysis, interpretation of data, the writing of this article or the decision to submit it for publication. The remaining authors declare that the research was conducted in the absence of any commercial or financial relationships that could be construed as a potential conflict of interest.

## Footnotes

1 https://imagej.net/Fiji

2 https://www.mipar.us/

## Supplementary Material

Supplementary Figure 1 Analysis of no–surgery blood vessels. Representative VVG images of contralateral, no–surgery femoral artery and vein from animals treated with PBS **(A)** and NVS **(B)**. C and D show IEL–enclosed area of the no–surgery vein **(C)** and no–surgery artery **(D)**, and compare them to the IEL–enclosed area of the AVF venous limbs and arterial limbs, respectively. Results are presented as average ± standard error of mean. N = 8–9 in each of the no–surgery artery and vein groups. * p < 0.001. ** p < 0.005. A = Artery. V = Vein. L = Lumen.

Supplementary Figure 2 Whole AVF and arterial limbs. Morphometric analysis of the whole AVF **(A–D)** and arterial limb **(E–H)** of the AVF anastomosis. Results are presented as average ± standard deviation. N = 3–4 per group in week 1. N = 6–9 per group in week 4.

Supplementary Figure 3 Analysis of collagen fibers in AVF. Representative multiphoton autofluorescence (green) and SHG (red) images of AVFs at 4 weeks from animals treated with PBS **(A,B)** and NVS **(C,D)**. B and D show the enlarged regions of interest used for the analysis of fiber structure.
