# Supplementary figures and images for: Natural Vascular Scaffolding Treatment Promotes Outward Remodeling During Arteriovenous Fistula Development in Rats
Source: Front Bioeng Biotechnol. 2021 Feb 15;9:622617. doi: 10.3389/fbioe.2021.622617 (PMC7928390; doi:10.3389/fbioe.2021.622617)

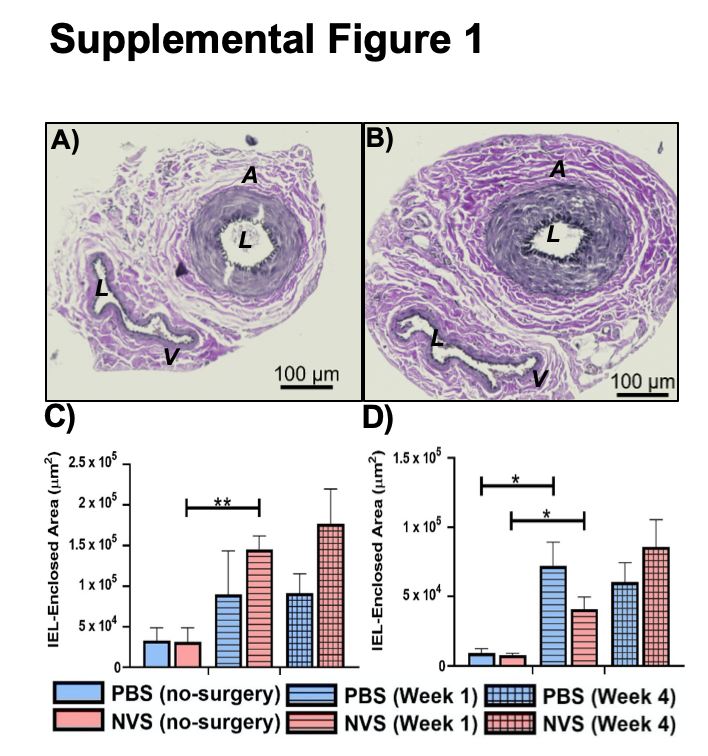

Supplement: Supplementary Figure 1 — Analysis of no–surgery blood vessels. Representative VVG images of contralateral, no–surgery femoral artery and vein from animals treated with PBS (A) and NVS (B). C and D show IEL–enclosed area of the no–surgery vein (C) and no–surgery artery (D), and compare them to the IEL–enclosed area of the AVF venous limbs and arterial limbs, respectively. Results are presented as average ± standard error of mean. N = 8–9 in each of the no–surgery artery and vein groups. * p < 0.001. ** p < 0.005. A = Artery. V = Vein. L = Lumen. [file Image_1.tiff]

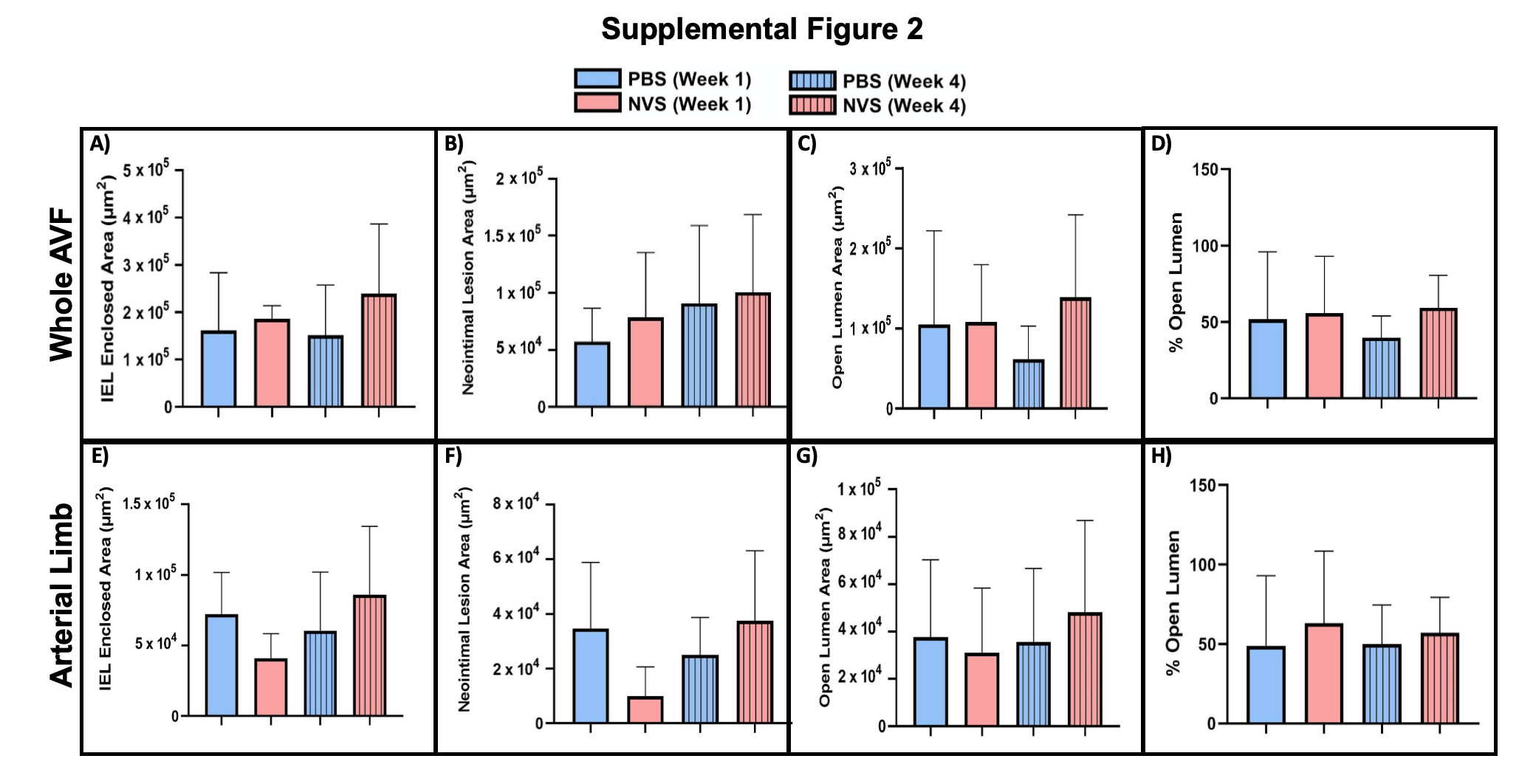

Supplement: Supplementary Figure 2 — Whole AVF and arterial limbs. Morphometric analysis of the whole AVF (A–D) and arterial limb (E–H) of the AVF anastomosis. Results are presented as average ± standard deviation. N = 3–4 per group in week 1. N = 6–9 per group in week 4. [file Image_2.tiff]

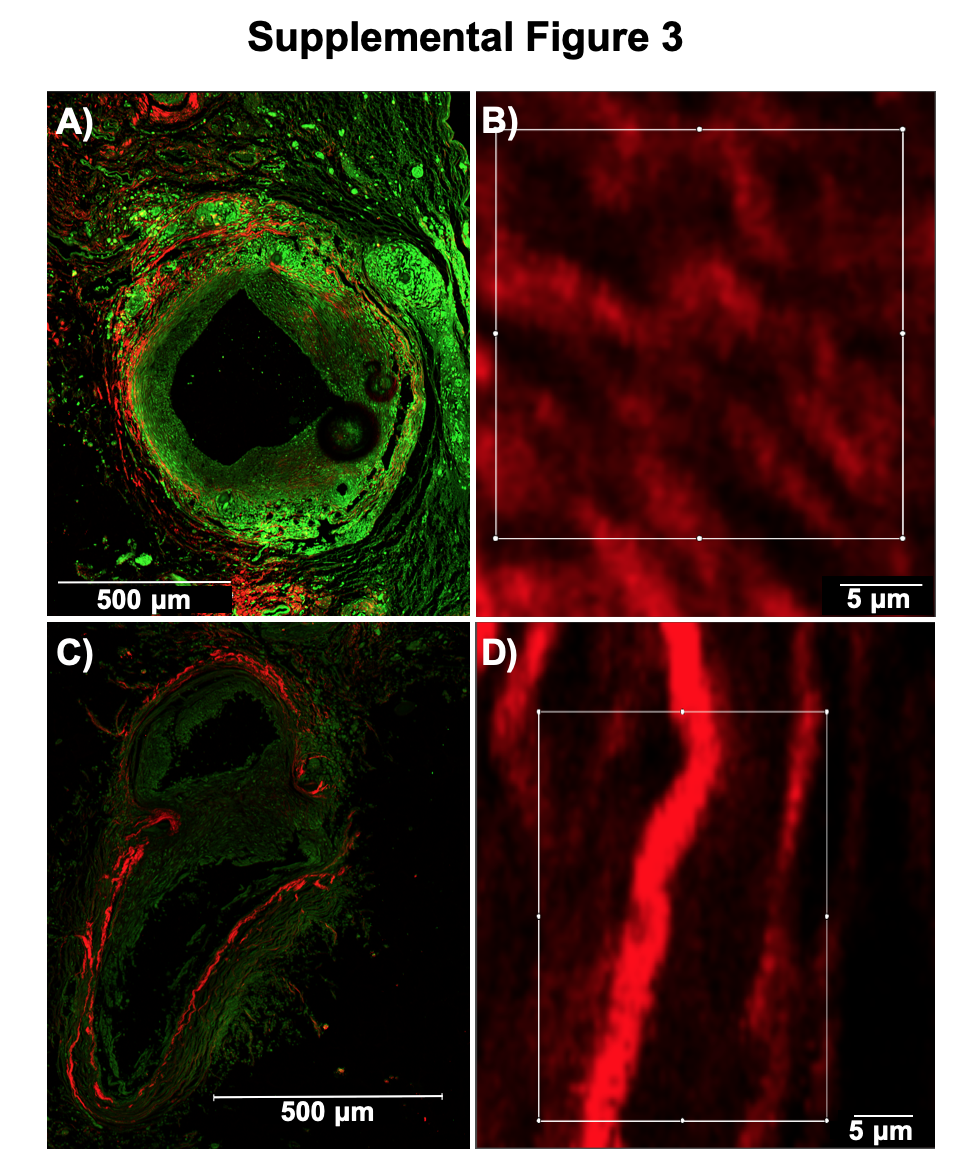

Supplement: Supplementary Figure 3 — Analysis of collagen fibers in AVF. Representative multiphoton autofluorescence (green) and SHG (red) images of AVFs at 4 weeks from animals treated with PBS (A,B) and NVS (C,D). B and D show the enlarged regions of interest used for the analysis of fiber structure. [file Image_3.tiff]
